# Effect of harness design for tag attachment on the flight performance of five soaring species

**DOI:** 10.1186/s40462-023-00408-y

**Published:** 2023-07-06

**Authors:** Arianna Longarini, Olivier Duriez, Emily Shepard, Kamran Safi, Martin Wikelski, Martina Scacco

**Affiliations:** 1 Department of Earth and Environmental Sciences, University of Pavia, Via Ferrata 1, 27100 Pavia, Italy; 2 Centre d’Ecologie Fonctionnelle et Evolutive, UMR 5175 CNRS-Université de Montpellier- EPHE-Université Paul Valery, 1919 Route de Mende, 34293 Montpellier Cedex 5, France; 3 Department of Biosciences, Swansea University, Swansea, SA2 8PP UK; 4 Max Planck Institute of Animal Behavior, Department of Migration, Am Obstberg 1, 78315 Radolfzell, Germany; 5 Department of Biology, University of Konstanz, Universitätsstraße 10, 78464 Konstanz, Germany; 6 Centre for the Advanced Study of Collective Behaviour, University of Konstanz, Universitätsstraße 10, 78464 Konstanz, Germany

**Keywords:** Tagging methods, Harness type, Backpack, Leg-loop, Soaring birds, Bio-logging, Flight performance

## Abstract

**Background:**

Bio-logging devices play a fundamental and indispensable role in movement ecology studies, particularly in the wild. However, researchers are aware of the influence that attaching devices can have on animals, particularly on their behaviour, energy expenditure and survival. The way a device is attached to an animal’s body has also potential consequences for the collected data, and quantifying the type and magnitude of such potential effects is fundamental to enable researchers to combine and compare data from different studies, as much as it is to improve animal welfare. For over two decades, large terrestrial birds have been in the focus of long-term movement ecology research, employing bio-logging devices attached with different types of harnesses. However, comparative studies investigating the effects of different harness types used on these species are scarce.

**Methods:**

In this study, we tested for potential differences in data collected by two commonly used harness types, backpack and leg-loop, on the flight performance of 10 individuals from five soaring raptor species, equipped with high resolution bio-logging devices, in the same area and time. We explored the effect of harness type on vertical speed, airspeed, glide ratio, height above sea level, distance travelled, proportion of soaring and flapping behaviour, and VeDBA (a proxy for energy expenditure) between and within individuals, all used as fine-scale measures of flight performance.

**Results:**

Birds equipped with leg-loops climbed up to 0.36 ms$^{-1}$ faster, reached 25.9% greater altitudes while soaring and spent less time in active flight compared to birds equipped with backpacks, suggesting that backpack harnesses, compared to leg-loops, might cause additional drag affecting the birds’ flight performance. A lower VeDBA, a lower rate of sinking while gliding and slightly higher glide ratio and airspeeds were also indicative of less drag using leg-loops, even though the effect on these parameters was comparable to inter-individual differences.

**Conclusions:**

Our results add to the existing literature highlighting the design-related advantages of leg-loops, and support the use of leg-loops as a better alternative to backpack harnesses for large soaring birds, when possible. Our study also highlights how apparently small changes in device attachment can lead to notable improvements in tagging practice, with implications for animal welfare, data interpretation and comparability.

**Supplementary information:**

The online version contains supplementary material available at (10.1186/s40462-023-00408-y).

## Background

The recent advances in the movement ecology field are sparked by the growing possibilities to remotely measure the movement and behaviour of animals in the wild. The use of bio-logging devices, such as GPS loggers, accelerometers and internal sensors, allow us to record an unprecedented amount of quantitative information concerning the movement and behaviour of an animal, its physiological condition and its environmental context [1].

Bio-logging techniques have a fundamental role in movement ecology studies, and the unprecedented frequency and accuracy of these data also provide us with new opportunities to compare bio-logging techniques and investigate their effect. Researchers are aware of the potential effects of bio-logging on animal behaviour and survival, and flying animals are in that respect of special concern. Although bio-logging is fundamental to study their long-distance movements, the added weight of a device can challenge their ability to remain aloft. In addition, the devices’ shape and position can increase drag during flight, and its attachment, when executed without the necessary diligence, create discomfort around the wings. In the last decade, meta-analyses reviewed the adverse effects of bio-logging on several important aspects of avian behaviour and ecology [2, 3], such as energy expenditure, survival rate, reproduction, parental care, foraging duration and speed [4]. However, other studies failed to find either short- or long-term differences in reproductive success, survival, activity budget and return rate at the colonies, attributable to the attachment of bio-logging devices [5–7].

Harnesses are indispensable for long-term bio-logging studies [8, 9]. Although large terrestrial birds, including many endangered species, are often the subject of such important research, studies investigating the effect of one or multiple harness types on these species are scarce, and usually based on few individuals (but see [10]). Long-term studies on raptors usually employ backpack-type (thoracic or wing-loop) harnesses [11, 12]. There is evidence that this type of harness in raptors causes irritation under the wings, physical discomfort and as a consequence increases preening behaviour [13–15]. Some studies showed that backpack harnesses decreased the survival in spotted owls *Strix occidentalis* [16] and prairie falcons *Falco mexicanus* [10], but another study did not find a long-term effect in black kites *Milvus milvus* [7]. The tightness and appropriate fit of the harness are of fundamental importance, as they can change over time with the growth of the animal or changes in its body conditions; hence the experience of the researcher attaching the harness is of primary importance. Birds equipped with backpacks are at risk of entangling their wings, especially if the harness is too loose. On the contrary, if too tight, this might inhibit the action of flight muscles or the deposition of fat [11, 17]. In more extreme cases, as reported by Peniche et al. [18] on red kites, the long-term attachment of backpacks can cause severe lesions and chronic inflammations. In addition, the design of backpack harnesses, consisting of two loops connected over the sternum, makes it difficult for the harness to fall off, in case of rupture of one of the loops. This would force the bird to unnecessarily keep carrying a damaged harness in an improper position and often failing to work (see however the new weak-link wing harness, suggested by Clewley et al. to ease detachment compared to permanent harnesses [19]).

**Fig. 1:**
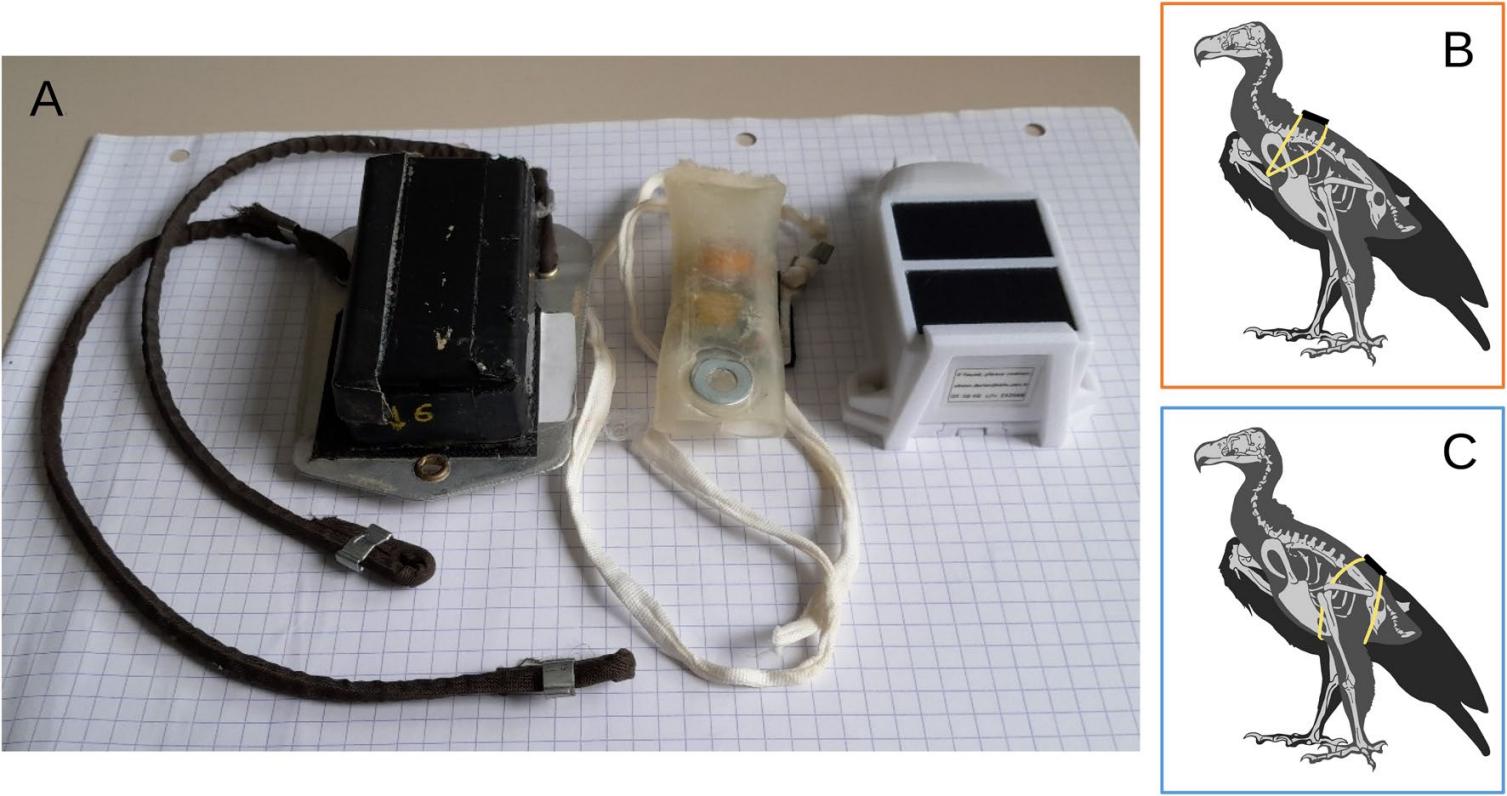
**A** Device cases + aluminium plates used in our study (*Gipsy* left, *Axytreck* middle) compared to an Ornitela OT50 device (right), commonly used in the field. In the right panel, schematic drawing of a backpack (“thoracic X-strap”, **B**), and a leg-loop harness (**C**). Harness illustrations by Louise Faure

Backpack harnesses are still widely used, particularly on terrestrial birds, and continue to provide indispensable insight into the movement of animals and their interactions with the environment, offering the basis for effective conservation and mitigation measures. However, alternative harness types deserve attention. In recent years, leg-loop harnesses (Rappole-type or pelvic harnesses [20]), widely used on passerines and shorebirds, have started being used on larger species too, especially seabirds [11]. Leg-loop harnesses consist of two loops, each passing around the bird’s thighs, with the device resting on its lower back (Fig. 1). Their design leaves wings, flight muscles and major fat deposits untouched, and if one side of the harness gets damaged, a leg-loop harness will easily fall off, reducing the risk of entanglement. Elastic leg-loops, despite certainly also representing a burden on the studied individuals, might therefore be considered a valid alternative to backpack harnesses. However, and this is important to highlight, the applicability of leg-loops is not universal, as for species with short thighs it is not a safe attachment method [8]. In addition, their design forces the device in a position that, compared to backpack harnesses, is further away from the bird’s centre of mass, which theoretically could cause higher energetic costs [21]. Biles et al. showed a lower return rate of American kestrels equipped with leg-loops compared to backpack [22], and Blackburn et al. discouraged the use of tied (*vs* elastic) leg-loop harnesses as they reduced re-sighting rate [23]. Also, due to the position of the device on the lower back, one study reported difficulties in solar-charging the battery attached with leg-loop design [11]. Therefore as for backpack harnesses, the applicability of leg-loops has to consider the morphological, demographic, and behavioural specifics of the species studied, with the goal of minimising impact on the natural behaviour of the individuals as an ethical responsibility, while also maximizing data quality and acquisition.

A very recent study from Mizrahy-Rewald et al. [24] on wild northern bald ibises showed that during migration, individuals equipped with leg-loops travelled significantly longer daily distances than individuals equipped with backpacks, and suggested that wearing a leg-loop rather than a backpack could potentially reduce the duration of their migration by 15% [24]. The same study also provided anecdotal evidence of backpack harnesses causing a constant flapping of the feathers along the bird’s back behind the device, indicating the presence of turbulence and increased drag.

The contradicting results found in the literature associated to the use of these two harness types calls for a better understanding of their impact at different scales. In particular, very few studies investigated the effect of the use of bio-logging on fine-scale flight performance, and none of them explicitly compared the effect of backpack *vs* leg-loop harnesses. In this study, we tested the effects of backpack and leg-loop harnesses on the fine-scale flight performance of 10 individuals from five soaring raptor species, equipped with high resolution bio-logging devices. Specifically, we explored the effect of using backpack *vs* leg-loop attachment on vertical speed, airspeed, glide ratio, height above sea level (a.s.l.), distance travelled, proportion of soaring and flapping behaviour, and VeDBA (Vectorial Dynamic Body Acceleration, a proxy for energy expenditure [25]), all used as measures of flight performance. The species involved were: griffon vulture (*Gyps fulvus*), Rüppell’s vulture (*Gyps rueppelli*), Himalayan griffon vulture (*Gyps himalayensis*), tawny eagle (*Aquila rapax*) and black kite (*Milvus migrans*). These five species are characterised by different morphologies, spanning a range of body masses from 0.8 to 8.4 Kg and wing spans from 1.38 to 2.8 m. The study was performed in a falconry park during a week of data collection, consisting of three flight sessions per day. During each flight session, we equipped the birds with high resolution GPS and accelerometry devices. The falconry park provided the unique setting of a common-garden experiment: all 10 individuals from the five species flew simultaneously in the same area, thus experiencing roughly the same environmental conditions; this minimized confounding factors related to the environmental context and facilitated comparisons across species. It also allowed us, during subsequent days, to collect data on the same individuals while attaching devices on them with one or the other harness type. This helped minimizing differences in flight performance related to the individuals’ behaviour rather than on the harness type. Moreover, all individuals were used to be handled on a daily basis, which likely reduced the stress usually associated with handling wild birds.

## Methods

### Data collection

The work was conducted in Rocamadour, France, at the *Le Rocher des Aigles* falconry centre (44.801962$^{\circ }$ N, 1.612855$^{\circ }$ E). This study site overlooks a 120 m-deep canyon, providing natural soaring conditions for raptors. Each animal, trained with falconry techniques for the public shows, was released from their perch and flew freely three times a day (at 10:00, 12:00 and 14:00, local time). After their release, the birds usually took-off immediately and had the possibility to fly for about 1 h (with an average flight duration of 41 min) to a maximum distance of 12.8 Km from the releasing point [764.9 m ± 29.4 (mean ± st.err.)]. Between the 25th of June and the 1st of July 2018, we collected GPS and tri-axial accelerometry (ACC) data on 10 individuals from five soaring raptor species: Eurasian griffon vulture (n=4), Rüppell’s vulture (n=1), Himalayan griffon vulture (n=2), tawny eagle (n=2) and black kite (n=1). During each flight, we recorded the time of departure and return of each individual to later isolate only GPS and ACC data collected during the flight sessions.

### Devices and harness types

We used GPS-ACC devices (Technosmart, IT) of different generations. Some devices had GPS and accelerometer sensors separated into two units: *Gipsy 1* (n=6) and *Gipsy 5* (n=1) recorded GPS locations at 4 Hz, and were associated with either *AXY 1* (n=4) or *AGM* (n=3) sensors, which collected ACC data at 25 Hz. Finally *Axytreck* devices (n=3) collected both 1 Hz GPS and 25 Hz ACC. *AXY 1* and *AGM* sensors were combined with either a *Gipsy 1* or a *Gipsy 5* sensor, in a plastic case of 7.5*4*2.2 cm (length, width and height respectively); while the plastic case hosting the *Axytreck* devices was smaller (8*2.6*1.3 cm) (Fig.  1A). Both types of plastic cases were fastened with Velcro on a small aluminium plate (of height 0.3 cm). The front cross-sectional area of the two combinations of devices and aluminium plates was 10 cm$^2$ for the larger devices and 4.16 cm$^2$ for the smaller *Axytreck*, corresponding to a range of 3 to 7% of the body cross-section of the study species.

The devices were attached to the birds’ body using a Teflon-nylon harness. Harness, aluminium plate and device were removed from the birds at the end of each day. The total weight of transmitter, aluminium plate and harness was 90 g for all the devices, except the one fitted on the black kite (*Axytreck*) and the *Gipsy 5* used in only one flight session, which had a total weight of 15 g and 60 g, respectively (including plate and harness). The weight of the equipment, relative to the birds’ body mass, ranged between 1.0% and 1.8% for all species except the tawny eagle. For the tawny eagle a smaller device was not available, and the weight of the equipment we applied corresponded to 3.2% and 3.9% of the body mass of the two individuals (Additional file 1: Table S1). The shape and weight of the largest case of our devices are comparable to those of an Ornitela GPS-GSM OT50 device, commonly used in field studies (height = 2.5 cm, weight device + harness = 60 g, frontal cross-section = 10 cm$^2$) (Fig. 1A).

The harness was fitted to the birds either as a leg-loop or as a backpack. Backpack harnesses were looped around the bird’s wings with the two loops crossing on the sternum, and the device positioned on the animal’s back between the scapulae. Leg-loop harnesses were looped around the bird’s thighs and the device positioned on the animal’s lower back. Both harness types are shown in Fig.  1BC and described in detail by Anderson et al. as “thoracic X-strap” harness and “leg-loop” (page 13 n. 1 and page 15 n. 9, respectively) [12].

All devices recorded GPS and ACC information continuously. At the beginning of each day, all tags were positioned on a wooden slat to be switched on and were calibrated simultaneously.

#### Validation data

We collected a validation dataset to assess if, for the same given behaviour, the position of the device on the animal’s back could affect the information we collected. To test for such differences we used data collected from one Eurasian griffon vulture during one day. During that day and two flight sessions, this individual was equipped simultaneously with both backpack and leg-loop attached devices. Both devices measured the same behaviour at the exact same time, and the GPS and ACC devices deployed were of the same generation (*Gipsy 1* and *AXY 1*). Therefore, we expect that potential differences between the flight parameters measured using the two harness types should be purely methodological and associated to the position of the device on the animal’s body.

#### Experimental group

The experimental group included 10 individuals. During each flight session, we equipped each individual with either a leg-loop or a backpack harness, randomizing the combination of device and harness type associated to each individual, to disentangle potential effects associated to the device type, the harness type and the individual behaviour. At the end of the study period and across different flight sessions, each individual bird could thus experience both types of attachment combined with different devices. Thus, each flight session of the day was considered as a separate unit and during each flight session, individuals were equipped with either a leg-loop or a backpack harness.

### Data processing and behavioural segmentation

The original dataset included 10 individuals from five species and a total of 96 flight sessions (40 with backpacks and 56 with leg-loops). Within each flight session, ACC and GPS data were recorded continuously. ACC data were collected at 25 Hz; GPS data at 1 and 4 Hz depending on the device generation, but they were all sub-sampled to 1 Hz (one GPS fix per second).

We used ACC data to identify active flight. We first calculated the static component of acceleration by taking running means (smoothed values) of the raw acceleration values of each of the three axis over a period of 0.5 s, corresponding to two complete flapping cycles (we observed an average of four flapping cycles per second) [26]. We then obtained the dynamic component of acceleration by subtracting the smoothed values from the raw values. We finally used the dynamic acceleration of the three axes to derive the VeDBA (Vectorial Dynamic Body Acceleration) [25, 27]. We averaged the VeDBA values per second and applied a k-means clustering algorithm with k=2 to distinguish between active flight (flapping) and passive flight (soaring or gliding, without flapping their wings). Average VeDBA values and activity classes were then associated to the GPS location matching in time.

To segment the GPS data, we applied a running mean of 15 s on the vertical speed; we then applied k-means clustering with k=2 on the smoothed vertical speed to distinguish soaring (positive vertical speed) from gliding behaviour (negative vertical speed). Vertical speed, horizontal speed and step length between consecutive GPS fixes were calculated for each flight session separately using the R package move [28].

The results of the two k-means clusterings, the one based on the smoothed VeDBA and the one based on the smoothed vertical speed, were finally combined in one variable with four classes: passive soaring, passive gliding, active soaring and active gliding. The results of the segmentation procedure were inspected visually by plotting the raw ACC values of the three axes and the GPS trajectories in three dimensions.

### Datasets

We analysed the effect of harness type on the flight parameters measured at two different levels.

We first focused on the level of the behavioural segment: consecutive GPS fixes belonging to the same behavioural class were assigned to the same segment ID. Only classified segments containing at least 5 consecutive fixes were kept in the dataset. Each entry of the dataset used in the analysis corresponded to one behavioural segment with the following associated parameters: mean vertical speed, mean horizontal speed, maximum height above sea level (a.s.l.), mean VeDBA, glide ratio (ratio between the distance covered in the horizontal plane and the distance dropped in height during each gliding segment) and airspeed (speed of the animal independent of the wind vector acting on it). Airspeed was calculated from the mean horizontal speed of the bird and the wind vector following Safi et al. [29]. The centroid of each behavioural segment was associated to the wind vector available at the closest time, location and height a.s.l. using the Env-DATA Track Annotation service in Movebank [30]. The associated wind data (U and V wind components) are available hourly at about 30 km resolution and were provided by the ECMWF Global Atmospheric Reanalysis ERA5 [31]. This dataset included both the validation data and the experimental group. The validation data included a total of 37 observations (behavioural segments, 18 backpack and 19 leg-loop) from 1 individual; all behavioural segments in the validation data were classified as passive behaviour (either soaring or gliding). The experimental group included 10 individuals and 2135 observations (789 backpack and 1346 leg-loop), of which only 62 were classified as active (flapping) flight.

We then worked at the level of the flight session. Each observation of this dataset corresponded to one flight session, whose performance was summarised in terms of: total flight duration, total distance covered during the flight, proportion of soaring flight along the track, proportion of active flight and cumulative VeDBA. The experimental group included 92 observations (flight sessions); data from the validation individual were excluded from this dataset, as the bird was only tracked for two flight sessions.

### Analysis of the behavioural segments

The average horizontal speed associated to the segments included in the analysis had a bi-modal distribution, with medians at 0.35 ms$^{-1}$ and 11.40 ms$^{-1}$, and a clear natural divide at 4 ms$^{-1}$. We thus used a 4 ms$^{-1}$ threshold to separate low from high speed segments [max. speed in low speed segments: 3.28 ms$^{-1}$; min. speed in high speed segments: 4.59 ms$^{-1}$]. The segments associated to very low speeds occurred during flight and could not be associated to a specific behaviour. For the following analysis we therefore considered only high speed segments (with average horizontal speed > 4 ms$^{-1}$).

Validation and experimental groups were analysed separately.

For the individual included in the validation dataset, we used two-sided Wilcoxon signed rank tests to assess if the differences in mean vertical speed, airspeed, glide ratio, maximum height a.s.l. and mean VeDBA measured using the two harness types was significantly different from 0.

For the experimental group, we used linear mixed-effects models (LMM) (R package lme4) [32] to test the effect of harness type on the flight performance parameters measured at the level of the flight segments. Mean vertical speed, airspeed, glide ratio, maximum height a.s.l. and mean VeDBA were used as response variables. As vertical speed and airspeed are known to differ between the soaring and gliding phases, we tested each of these two flight parameters separately, once during soaring and once during gliding. In contrast, as both soaring and gliding phases are expected to result in a similarly low activity level of VeDBA, we ran only one model for all passive flight segments testing for differences in VeDBA between harness types. Active flight segments (62 observations in total) were excluded from the VeDBA model. Maximum height a.s.l. was only analysed for soaring segments, while glide ratio was only analysed for gliding segments. We found unrealistically high glide ratios (between 100 and 914)) to be associated with very low sinking rate (mean vertical speed > $-$0.16 ms$^{-1}$, more similar to horizontal flight than gliding); we therefore excluded these observations (68 out of 927) and included in the glide ratio model only gliding segments with vertical speed < $-$0.2 ms$^{-1}$.

In all seven models, harness type and species were included as interacting categorical predictors, to account for potential differences in the way the different species were affected by the two harness types. Using ANOVA, we assessed the statistical significance of the interaction term and of the harness type, by comparing the full model with null models not including these terms. Hour of the day (with 0 centered at 12:00 UTC) was also included as predictor in all models to acknowledge changes in flight parameters at different times of the day. Finally, we included the segment length (number of fixes in the segment) to account for the variability in the duration of the behavioural segments. Date of the flight session and individual identity were included as random terms in all models.

In order to reduce temporal auto-correlation, the models predicting airspeed, height a.s.l. and VeDBA were run on a subset of the dataset, including one every second, one every fourth and one every third observation respectively. The response variable glide ratio was square-root transformed while the variables height a.s.l. and VeDBA were log transformed and all models were fitted with a Gaussian error distribution.

### Analysis of the flight sessions

We used non-parametric Wilcoxon tests on the experimental individuals to compare the measured flight parameters between harness types. Specifically, for each species $\alpha$ and for each flight parameter *P*, we computed the absolute difference between all combinations of observations of backpack (*BP*) and leg-loop (*LL*). This difference was defined as:

$$\Delta P^{\alpha } = | P_i^{\alpha ,BP} - P_j^{\alpha ,LL}|$$,

where *i* and *j* represent the *i*th and *j*th observation (flight session) associated to each harness type. To avoid replicates, we ensured that the number of observations was equal between the two groups: when the number of observations was higher for one of the two harness types, we randomly sub-sampled the number of observations associated to the second harness type.

We then tested whether the distribution of absolute differences between the groups ($\Delta P^{\alpha }$) was higher (one-sided Wilcoxon test) than the mean of absolute differences within groups (baseline). The baseline *B* was defined as:

$$B = {\bar{X}}( | P_i^{\alpha ,H} - P_j^{\alpha ,H}| )$$,

where *H* represents the respective harness type and $\alpha$ the species, as the baseline was calculated within species and within harness type.

Data processing and analysis were performed in R [33]. The complete R scripts used for the analysis are available at https://doi.org/10.5281/zenodo.7742121

## Results

### Analysis of the behavioural segments

Our data included a total of 2172 observations (behavioural segments) including both validation and experimental data (see also Additional file 1: Table S1)).

#### Validation data

Using the two-sided Wilcoxon tests we detected no significant difference in the distribution of the five flight parameters between backpack and leg-loop segments, indicating that the accuracy of the information measured by the devices was not affected by their position [mean vertical speed: W = 170.5, *p* = 1; airspeed: W = 129, *p* = 0.21; glide ratio: W = 27, *p* = 0.75; maximum height a.s.l.: W = 180.5, *p* = 0.78; mean VeDBA: W = 146, *p* = 0.46].

#### Experimental group

**Fig. 2:**
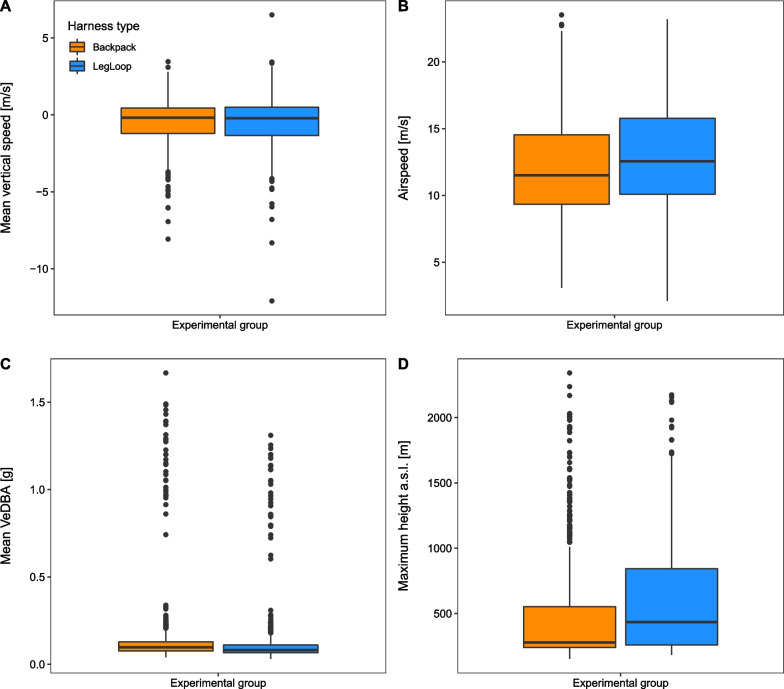
Overview of the linear magnitude of the difference in the flight parameters considered, between harness types and across all flight behaviours: **A** Average vertical speed, **B** airspeed, **C** average VeDBA, and **D** maximum height a.s.l. calculated per behavioural segment. Different colours differentiate between individuals equipped with backpack and leg-loop harnesses

**Fig. 3:**
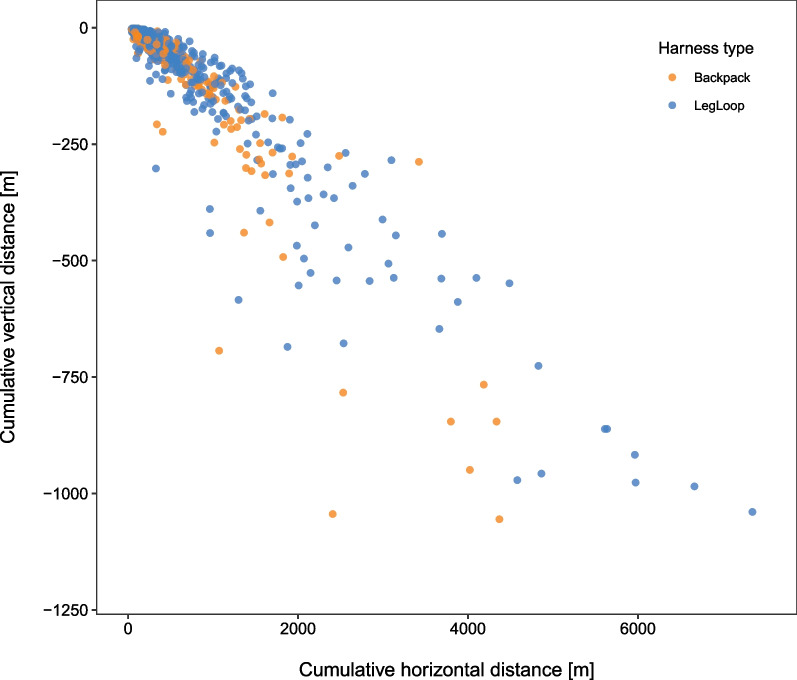
Cumulative distance covered in the horizontal plane relative to the cumulative vertical distance dropped per gliding segment. Different colours differentiate between individuals equipped with backpack and leg-loop harnesses

The distribution of the five flight parameters associated to each behavioural segment (mean vertical speed, airspeed, glide ratio, maximum height a.s.l. and mean VeDBA) relative to the harness type is shown in Figs. 2 and 3. All models’ results listed below, unless otherwise specified, show estimate ± st.err.

**Table 1:** Output of the LMM with mean vertical speed included as dependent variable, number of fixes, hour of the day, harness type and species as fixed terms, individual identity and date as random intercepts. The interaction term between harness type and species was non significant in the gliding model and therefore excluded

	Soaring segments	Gliding segments
**Fixed effects** **estimate (St. Err.)**		
Intercept	0.39 (0.15)*	−0.99 (0.33)*
Leg-loop	−0.29 (0.19)	0.15 (0.08)*
Tawny eagle	−0.18 (0.19)	−0.71 (0.43)
Griffon vulture	−0.22 (0.15)	−0.40 (0.37)
Himalayan vulture	−0.20 (0.16)	−0.33 (0.40)
Rüppell’s vulture	−0.50 (0.17)*	−0.85 (0.46)
Hour	−0.08 (0.02)***	0.02 (0.03)
Number of fixes	0.004 (0.0002)***	−0.007 (0.0005)***
Leg-loop*Tawny eagle	0.02 (0.26)	
Leg-loop*Griffon vulture	0.51 (0.20)*	
Leg-loop*Himalayan vulture	0.39 (0.21).	
Leg-loop*Rüppell’s vulture	0.65 (0.25)**	
**Random effects (N. groups)** **intercept St. Dev.**		
Individuals	0.05 (10)	0.30 (10)
Date	0.16 (7)	0.15 (7)
Observations	1208	926
Marginal R$^2$	0.25	0.19
Conditional R$^2$	0.29	0.28

$$p<$$0.1; *$$p<$$0.05; **$$p<$$0.01; ***$$p<$$0.001

In the vertical speed model associated to soaring, the effect of harness type differed between species, the interaction term being significant compared to the null model [$\chi ^2$ = 15.17, *p* = 0.004]. All vultures species equipped with leg-loops reached significantly higher vertical speeds while soaring, up to 0.65 ms$^{-1}$ higher (Rüppell’s vulture), compared to the backpack group [leg-loop:Griffon vulture = 0.51 ± 0.20; leg-loop:Himalayan vulture = 0.39 ± 0.21; leg-loop:Rüppell’s vulture = 0.65 ± 0.25], while the effect on the black kite and the tawny eagle was statistically non significant (Table 1). In the gliding model the effect of harness type did not differ between species [$\chi ^2$ = 4.99, *p* = 0.29] but overall all species showed a significant increase in vertical speed (lower sinking rate) when equipped with leg-loops [leg-loop = 0.15 ± 0.08] (Table 1).

**Table 2:** Output of the LMM with airspeed included as dependent variable, number of fixes, hour of the day, harness type and species as fixed terms, individual identity and date as random intercepts. The interaction term between harness type and species was non significant in the gliding model and therefore excluded

	Soaring segments	Gliding segments
**Fixed effects** **estimate (St. Err.)**		
Intercept	6.62 (0.74)***	10.02 (1.03)***
Leg-loop	0.41 (0.27)	0.21 (0.38)
Tawny eagle	2.11 (0.85)*	1.51 (1.38)
Griffon vulture	5.32 (0.72)***	4.63 (1.10)**
Himalayan vulture	4.90 (0.73)***	3.60 (1.14)*
Rüppell’s vulture	3.94 (0.81)**	3.88 (1.32)*
Hour	−0.07 (0.1)	−0.001 (0.13)
Number of fixes	0.001 (0.001)	0.01 (0.003)***
**Random effects (N. groups)** **intercept St. Dev.**		
Individuals	0.38 (10)	0.76 (10)
Date	0.94 (7)	0.96 (7)
Observations	604	464
Marginal R$^2$	0.18	0.15
Conditional R$^2$	0.27	0.25

$$p<$$0.1; *$$p<$$0.05; **$$p<$$0.01; ***$$p<$$0.001

In the case of the airspeed, in both the soaring and the gliding models the effect of harness type did not differ between species [soaring: $\chi ^2$ = 1.53, *p* = 0.82; gliding: $\chi ^2$ = 5.71, *p* = 0.22]. In both models, all individuals showed a slight increase in airspeed when equipped with leg-loops; they were predicted to fly 0.41 ms$^{-1}$ faster when soaring and 0.21 ms$^{-1}$ faster when gliding, although in both cases this effect was not statistically significant (Table 2).

**Table 3:** Output of the LMM with the square root of the glide ratio included as dependent variable, number of fixes, hour of the day, harness type and species as fixed terms, individual identity and date as random intercepts. The model included only gliding segment with vertical speed < 0.2 ms^−1^. The interaction term between harness type and species was not significant and therefore excluded

	Gliding segments
**Fixed effects** **estimate (St. Err.)**	
Intercept	3.35 (0.20)***
Leg-loop	0.16 (0.08)*
Tawny eagle	−0.41 (0.28)
Griffon vulture	0.09 (0.22)
Himalayan vulture	0.003 (0.23)
Rüppell’s vulture	−0.30 (0.26)
Hour	0.009 (0.03)
Number of fixes	-0.005 (0.0005)***
**Random effects (N. groups)** **intercept St. Dev.**	
Individuals	0.15 (10)
Date	0.16 (7)
Observations	859
Marginal R$^2$	0.12
Conditional R$^2$	0.16

s$$^{-1}$$.

$$p<$$0.1; *$$p<$$0.05; **$$p<$$0.01; ***$$p<$$0.001

In the glide ratio model the effect of harness type did not differ between species [$\chi ^2$ = 2.52, *p* = 0.64] but overall, birds equipped with leg-loops showed a small and slightly significant increase in glide ratio [leg-loop = 0.16 ± 0.08]. This translates in about 1.07 m increase in horizontal distance covered per meter of drop for birds wearing leg-loops (Table 3; please note that the variable glide ratio was square-root transformed, and the estimates interpreted accordingly).

**Table 4:** Output of the LMM with the log of the maximum height a.s.l. included as dependent variable, number of fixes, hour of the day, harness type and species as fixed terms, individual identity and date as random intercepts. The model included only soaring segments and was run on a subset of the dataset (one every fourth observation was kept) to reduce temporal auto-correlation. The interaction term between harness type and species was non significant and therefore excluded

	Soaring segments
**Fixed effects** **estimate (St. Err.)**	
Intercept	5.28 (0.16)***
Leg-loop	0.23 (0.001)***
Tawny eagle	0.27 (0.19)
Griffon vulture	0.56 (0.16)**
Himalayan vulture	0.47 (0.16)*
Rüppell’s vulture	0.34 (0.17).
Hour	−0.03 (0.02)
Number of fixes	0.0002 (0.0002)***
**Random effects (N. groups)** **intercept St. Dev.**	
Individuals	0.06 (10)
Date	0.17 (7)
Observations	302
Marginal R$^2$	0.30
Conditional R$^2$	0.37

$$p<$$0.1; *$$p<$$0.05; **$$p<$$0.01; ***$$p<$$0.001

In the model predicting the maximum height a.s.l. during soaring the effect of harness type did not differ between species, the interaction term being non significant compared to the null model [$\chi ^2$ = 5.16, *p* = 0.27]. The model showed that birds equipped with leg-loops reached higher altitudes during soaring. This effect was highly significant and associated to a 25.9% increase in maximum height a.s.l. [leg-loop = 0.23 ± 0.001] (Table 4; please note that height a.s.l. was log transformed).

**Table 5:** Output of the LMM with the log of the mean VeDBA included as dependent variable, number of fixes, hour of the day, harness type and species as fixed terms, individual identity and date as random intercepts. The model included only passive flight and was run on a subset of the dataset (one every third observation was kept) to reduce temporal auto-correlation. The interaction term between harness type and species was non significant and therefore excluded

	Passive segments
**Fixed effects** **estimate (St. Err.)**	
Intercept	−1.78 (0.11)***
Leg-loop	−0.09 (0.03)***
Tawny eagle	−0.16 (0.14)
Griffon vulture	−0.55 (0.12)*
Himalayan vulture	−0.65 (0.13)*
Rúppell’s vulture	−0.54 (0.15).
Hour	−0.01 (0.009).
Number of fixes	0.0006 (0.0001)***
**Random effects (N. groups)** **intercept St. Dev.**	
Individuals	0.10 (10)
Date	0.07 (7)
Observations	691
Marginal R$^2$	0.29
Conditional R$^2$	0.38

$$p<$$0.1; *$$p<$$0.05; **$$p<$$0.01; ***$$p<$$0.001

Finally, also in the model predicting mean VeDBA the interaction term between harness type and species was not significant [$\chi ^2$ = 3.74, *p* = 0.44]. Overall, all birds showed a statistically significant decrease in VeDBA ($-$9.9%) when equipped with a leg-loop compared to a backpack [leg-loop = $-$0.09 ± 0.03] (Table 5, note that the variable mean VeDBA was log transformed).

In two of the five flight parameters investigated (vertical speed during soaring and height a.s.l.), the effect size associated to the harness type was much higher than the among-individuals and among-dates variability (intercept standard deviation) (Tables 1,2,4). This suggests that the statistically significant variance which we found associated with the harness type, at least in these two flight parameters, could be relevant from a biological perspective.

### Analysis of the flight sessions

We applied one-sided Wilcoxon test (greater) and found that the difference in flight parameters between harness types was never significantly higher than the baseline, except in the case of the proportion of active flight. In this case, the difference in the proportion of active flight performed with one or the other harness type was significantly higher than the baseline [one-sided Wilcoxon test: V = 40456, *p* = 0.0002]; the mean of the difference between groups was positive, meaning that birds wearing backpacks spent a higher proportion of time using active flight compared to birds wearing leg-loops.

## Discussion

In this study we compared the effect of leg-loop and backpack harnesses on the flight performance of 10 individuals from five soaring raptor species, in a unique setting that allowed us to minimize confounding factors related to environmental context, individual behaviour and handling stress. To our knowledge, this is the first comparison of the effect of two harness types on the fine-scale flight performance of multiple species. During the analysis we accounted for the animal’s flight behaviour, and analyzed flight performance at the scale of the behavioural segments as well as at the scale of the flight session.

At the level of the behavioural segment, the validation individual showed no difference in the flight parameters collected simultaneously by the two harness types, showing that the information we collected were not likely to be affected by the positioning of the device on the animal’s back. The results of the models investigating the effect of harness type on the experimental individuals showed differences in flight performance associated to the two harness types, that might suggest a lower drag associated with leg-loop compared to backpack harnesses. We could not directly manipulate the effect of drag and its different components; hence in this study we use the word “drag” to refer to any added flight cost, as an increase in actual parasite drag or a loss of lift due to flow interruption. We also couldn’t disentangle the potential effect of factors other than drag on our results, such as a restriction to the action of the flight muscles. However, the parameters affected and the magnitude of the differences found, were largely consistent with drag being the main explanation behind these differences, begging for additional experimental investigations into the precise interaction between tag placement and flight performance. In particular, our data showed that birds equipped with leg-loops climbed up to 0.36 ms$^{-1}$ faster and reached 25.9% higher altitudes while soaring. A decreased drag associated with the use of leg-loops was also suggested by a lower rate of sinking while gliding and a slightly higher glide ratio, both suggesting that birds equipped with leg-loops could cover a higher horizontal distance per unit of drop in height. Birds wearing leg-loops also showed slightly higher airspeeds and 9.9% lower VeDBA. The latter indicates a lower degree of movement either for the tag or for the animal, both of which suggest lower energetic expenditure. The variability of these last two parameters associated to the use of leg-loops was comparable to the inter-individual variability, suggesting that the observed difference in airspeed and VeDBA between the two harness types might not be biologically relevant.

At the level of the flight session, birds wearing leg-loops seemed to spend less time using active flight compared to individuals wearing backpacks, but no other differences were detectable in any of the other flight parameters. A lower proportion of active flight should correspond to a lower energy expenditure during the flight session, although we did not find any difference in cumulative VeDBA between harness types.

Overall, most of our results showed lower flight performance associated with the use of backpack harnesses, probably as a consequence of additional drag caused by the device in its position. This is consistent with a study that visualised the flow over a model penguin, which demonstrated that device-induced turbulence was lower when loggers were placed further back on the body, specifically after the point with maximum girth, where the boundary layer becomes turbulent [34]. In our study, the suggested reduction in drag associated with the leg-loop harness resulted in a substantial improvement in flight performance compared to birds with backpacks. For instance, the increase in vertical speed for griffon vultures equipped with leg-loops (0.51 ms$^{-1}$ higher compared to backpacks) represents $\sim$45% of the average vertical speed reported for this species soaring in Israel (1.1 ms$^{-1}$ [35]). It should be clear that this could make a substantial difference to the overall cross country speed of these birds given the time they spend in soaring flight (birds in Israel undertook 22.8 thermal soaring cycles per day [35]), even before the improvements in airspeed and glide ratio are factored in.

The effects we found on the fine-scale flight performance of these species complement the larger scale findings of a very recent study from Mizrahy-Rewald et al. on migrating northern bald ibises, showing that birds wearing backpacks migrated shorter daily distances than birds wearing leg-loops [24]. We note that other considerations may also affect the optimal logger location, as this at the least affects the centre of gravity [21]. This is less likely to be an issue for large birds, such as those in this study, where loggers constitutes a small fraction of their body mass, but should be taken into account when considering the use of leg-loops on smaller species.

Our results showed that minute difference in the position of the device on the animal’s back could impact the birds’ flight performance. Although in our experimental setup the tags were attached on an aluminium plate, causing potentially more lift than necessary from the animal body and exacerbating the detrimental effects found, the stark differences that arose from the placement of the tags remain indisputable. Cases of small changes producing a surprisingly large impact are already reported in the literature. For instance, Saraux et al. [36] showed that flipper bands on penguins had population-level impacts in some scenarios, associated with an increase in energy expenditure; and Pennycuick [37] showed that a 6 mm high transmitter increased the drag coefficient of a rose-coloured starling by an estimated 50%. The large impact that small changes in device shape and position can potentially cause should encourage our research community to invest more in studying the effect of device attachment.

In the last 25 years, several studies highlighted side effects of backpack harnesses on terrestrial bird species [8, 10, 13, 14, 16]. Our results add to the existing literature in support of considering leg-loops as a good alternative to backpack harnesses, at least for relatively large species. In addition to the positive effect (relative to backpacks) on the birds’ flight performance, suggested by our results, the design of leg-loops has other clear advantages. Leg-loops leave wings, flight muscles and major fat deposits untouched [8, 11] and they reduce the risk of entanglement as, in case of damage, they fall off. Leg-loop harnesses are also faster to fit on birds, reducing handling time (especially important when handling wild species), and potentially their stress level. Finally, leg-loops require less material, hence reducing the overall weight of the harness.

Even though our analysis of flight performance supports the use of leg-loops, compared to backpack harnesses, the data used in this study are based on a limited period of data collection and captive individuals. We therefore could not investigate other important parameters such as change in the individual’s behaviour before and after equipping the animals with harnesses, nor potential long-term effects on the individuals’ reproductive success and survival. Such potential additional effects have to be investigated independently too, as they cannot be excluded based on results related to flight parameters only.

Experience gained with long-term studies using a specific harness type is also useful to evaluate technical improvement. One study, using leg-loops on seabirds, reported that due to the tag position on the animal’s back, the solar panel was covered by feathers and could not charge the device’s battery [11]. In our study we used devices without solar panels, and we could therefore not investigate such technical problems. However, we are aware of long-term tracking studies on griffon vultures and northern bald ibises using solar-powered tags fitted as leg-loops [24, 38–40] as well as a few other ongoing studies with large soaring raptors wearing leg-loop mounted GPS devices. We thus think that technical problems related to energy harvesting can be species specific and in many cases overcome, maybe even reduced through the mere use of leg-loops, at least within the limits posed by the local atmospheric conditions (e.g. hours of sun) and the species-specific behaviour (e.g. time spent flying) and plumage.

Investigating the effect of harness type on fine-scale flight parameters is also relevant in the context of data standardization and comparability [4]. The measures of flight performance investigated in our study are commonly used parameters in movement ecology studies focusing on comparing flight behaviour and performance across species, populations or environmental contexts. The data used in such studies are often collected by different research groups using different devices with possibly different attachment methods. It is therefore of primary importance to investigate how the methodology used to measure these information affects the collected data. Not only to the benefit of the animals’ welfare, but also to avoid systematic bias in our results, which would invalidate data comparability and lead to misinterpreting the behaviour we are trying to measure [3, 4].

## Conclusions

Bio-logging devices are indispensable in movement ecology research, but comparative studies investigating the effect of different device attachments are still rare. The available harness types differ in terms of the body parts they restrict, in how easily they can move or fall off, and in the resulting position of the device on the animal body, which can in turn affect the device’s drag. The results of our study shows that in large terrestrial species, leg-loop harnesses can be advantageous not only in terms of their design but also because of the likely reduced drag imposed on the birds, which results in improved fine-scale flight performance; in such species, leg-loops can therefore be considered as a good alternative to the commonly used backpack harnesses.

The awareness and quantification of the bias caused by different attachment types will not only benefit our study species, but also allow our research community to make best use of existing data and gain better and more complete insight into the movement ecology field, by using larger sets of data and taking advantage of the comparative aspect that meta-analyses can provide.

## Supplementary information

**Additional File 1.** Supplementary material containing Table S1, referenced in the Methods.

## Data Availability

The data that support the findings of this study and the R scripts used to process and analyse the data are available at https://doi.org/10.5281/zenodo.7742121

## Abbreviations

ACC: Tri-axial accelerometry
VeDBA: Vectorial Dynamic Body Acceleration
BP: Backpack harness
LL: Leg-loop harness
