# Supplementary material for: Effect of harness design for tag attachment on the flight performance of five soaring species
Source: Mov Ecol. 2023 Jul 6;11:39. doi: 10.1186/s40462-023-00408-y (PMC10326940; doi:10.1186/s40462-023-00408-y)
Supplement: Supplementary file 1 — Additional File 1. Supplementary material containing Table S1, referenced in the Methods. [file 40462_2023_408_MOESM1_ESM.pdf]

Effect of harness design for tag attachment on the flight performance of five soaring species

SUPPLEMENTARY MATERIAL

Contents

S1 Analysis of the behavioural segments **2**

S1 Analysis of the behavioural segments

Table S1: Overview of the body mass, the mass of the attached devices (including harness) relative to the body mass, and the number and duration of the behavioural segments included in the LMMs, per bird and harness type (*LL = leg-loop, BP = backpack*).

**Species**	**Bird ID**	**Body mass (kg)**	**Device mass / Body mass**	**N. segments**	**Mean segments duration (s)**
Black kite	Klea	0.83	0.018	60 (LL)<br>83 (BP)	40.6 (LL)<br>34.5 (BP)
Griffon vulture	Gregoire	7.60	0.012	187 (LL)<br>102 (BP)	60.8 (LL)<br>51.0 (BP)
Griffon vulture	Henri	7.55	0.012	199 (LL)<br>184 (BP)	42.7 (LL)<br>34.8 (BP)
Griffon vulture	Leon	6.80	0.013	106 (LL)<br>11 (BP)	49.7 (LL)<br>26.3 (BP)
Himalayan vulture	Gaelle	8.40	0.010	104 (LL)<br>89 (BP)	69.6 (LL)<br>42.7 (BP)
Himalayan vulture	Giselle	8.00	0.011	497 (LL)<br>110 (BP)	54.3 (LL)<br>46.3 (BP)
Rüppell's vulture	Kirikou	5.45	0.017	111 (LL)<br>154 (BP)	44.1 (LL)<br>77.2 (BP)
Tawny eagle	Manu	2.77	0.032	19 (LL)<br>56 (BP)	23.3 (LL)<br>22.5 (BP)
Tawny eagle	Khan	2.33	0.039	56 (LL)<br>0 (BP)	35.2 (LL)<br>0 (BP)
